# Assessment of skeletal muscle fatigue of road maintenance workers based on heart rate monitoring and myotonometry

**DOI:** 10.1186/1745-6673-1-20

**Published:** 2006-07-27

**Authors:** Zenija Roja, Valdis Kalkis, Arved Vain, Henrijs Kalkis, Maija Eglite

**Affiliations:** 1 Faculty of Chemistry, University of Latvia, Kr. Valdemara 48, Riga LV-1013, Latvia; 2 Institute of Experimental Physics and Technology, University of Tartu, Tahe 4, Tartu 51010, Estonia; 3 Faculty of Economics and Management, University of Latvia, Aspazijas bulv.5, Riga LV-1050, Latvia; 4 Institute of Occupational and Environmental Health, Riga Stradins University, Dzirciema 16, Riga LV-1007, Latvia

## Abstract

**Objective:**

This research work is dedicated to occupational health problems caused by ergonomic risks. The research object was road building industry, where workers have to work very intensively, have long work hours, are working in forced/constrained work postures and overstrain during the work specific parts of their bodies. The aim of this study was to evaluate the work heaviness degree and to estimate the muscle fatigue of workers after one week work cycle. The study group consisted of 10 road construction and maintenance workers and 10 pavers aged between 20 and 60 years.

**Methods:**

Physical load were analyzed by measuring heart rate (HR), work postures (OWAS) and perceived exertion (RPE). Assessments of the muscles strain and functional state (tone) were carried out using myotonometric (MYO) measurements. The reliability of the statistical processing of heart rate monitoring and myotonometry data was determined using correlating analysis.

**Results:**

This study showed that that road construction and repairing works should be considered as a hard work according to average metabolic energy consumption 8.1 ± 1.5 kcal/min; paving, in its turn, was a moderately hard work according to 7.2 ± 1.1 kcal/min. Several muscle tone levels were identified allowing subdivision of workers into three conditional categories basing on muscle tone and fatigue: I – absolute muscle relaxation and ability to relax; II – a state of equilibrium, when muscles are able to adapt to the work load and are partly able to relax; and III – muscle fatigue and increased tone. It was also found out that the increase of muscle tone and fatigue mainly depend on workers physical preparedness and length of service, and less – on their age.

**Conclusion:**

We have concluded that a complex ergonomic analysis consisting of heart rate monitoring, assessment of compulsive working postures and myotonometry is appropriate to assess the work heaviness degree and can provide prognosis of occupational pathology or work-related musculoskeletal disorders for the workers under different workload conditions. These results can also be used when deciding on necessary rest time and its periodicity.

## Background

It is well-known that work related diseases of muscle and skeletal system are prevalent all over Europe. In Latvia, where the amount of work and its intensity in the construction sector has substantially increased after joining the European Union in 2004, one of the main tasks of occupational health care is to prevent work related injuries. Similarly, despite mechanization the number of occupational diseases, as well as cumulative trauma disorders (CTD) caused by overwork, has rapidly increased in Latvia. They are caused by ergonomic factors of the work environment, such as physical overload, compulsive working postures, local stiffness of definite muscle groups and an adverse microclimate. CTD is not easy to diagnose and is difficult to treat; therefore, these disorders need to be prevented. Prevention is possible if the changes in the skeletal muscles causing CTD are identified as early as possible.

The aim of this study was to assess the work heaviness basing on heart rate monitoring and to assess muscle fatigue of workers after one week work cycle applying myotonometric method and special equipment to perform biomechanical diagnostics of functional state of skeletal muscles. This study deals also with the monitoring of workers' ability to adapt to intensive physical loads.

Workers from the road construction sector were chosen for this research because road maintenance and repair work is characterized by very variable work cycles with differing difficulty levels when specific works have to be performed.

For the study workers were selected following such criteria: full-time workers, no acute musculoskeletal symptoms, work experience of at least one year in the construction industry, and full consent to participate. The study plan was accepted by the Ethics Committee of the Paul Stradins University's Institute of Occupational and Environmental Health.

## Study group and methods

### Study design

One of the largest road building companies in Latvia with more than 600 employees was chosen. The examination was offered to workers whose work is characterized by variable work cycles and performance of specific works. Although the research involved more than 100 persons, more demonstrative results are shown and work heaviness, as well as changes of muscle tone are analyzed for those workers who work in groups (teams). The all-male group consisted of road workers (10) and pavers (10). All the workers were right-handed. Background factors of the subjects are shown in Table 1.

**Table 1:** Background factors of the subjects, mean, standard deviation (SD) and range

**Variable**	**Road workers (n = 10)**		**Pavers (n = 10)**	
	
	**Mean ± SD**	**Range**	**Mean ± SD**	**Range**
Age (years)	40 ± 4	20–60	30 ± 4	30–60
Height (cm)	180 ± 5	173–187	172 ± 7	165–180
Weight (kg)	81 ± 9	65–97	76 ± 6	60–92
Body mass index (BMI, kg/m^2^)	25 ± 6	17–36	25 ± 3	19–28
Rest heart rate (beats/min)	67 ± 7	56–78	62 ± 6	50–74

### Methods

The work heaviness degree depending on workers physical activity (intensity) was estimated by heart rate monitoring (HRM). The measurement was based on heart rate (HR) variation, which correlates with oxygen consumption and allows quantifying the objective energy expenditure for each work phases including short rest periods [1]. HRM was performed using *POLAR S810i*™ Heart Rate Monitor device and data processing software *Polar Precision Performance*. The device sums up the acquired HR data and transforms them into metabolic energy consumption (kcal/min). The relative range of the HR (%HRR) was calculated using a following equation: 100*{(HR_work_-HR_rest_)/(HR_max_-HR_rest_)}[2]. Maximal heart rate was calculated as the most common formula HR_max _= 220-age, although there exist most accurate formulas, for example: HR_max _= 205.8-(0.685*age) [3]. Work heaviness in terms of energy expenditure was classified according to classification scale shown in Table 2.

**Table 2:** Work heaviness classification in terms of energy expenditure

**Workload categories**				**Energy expenditure**	
**NIOSH (USA) standard **[4], **ISO 28996**		**Russian standards of hygiene **[5]		**Male, kcal/min**	**Female, kcal/min**

Light work	I	Light work	I	2.0 – 4.9	1.5 – 3.4
Moderate work	II	Permissible work	II	5.0 – 7.4	3.5 – 5.4
Hard work	III	Moderate work	II.1	7.5 – 9.9	5.5 – 7.4
Very hard work	IV	Hard work	II.2	10.0 – 12.4	7.5 – 9.4
Ultimate work	V	Excessively hard work	III	more 12.5	more 9.5

The work postures were analysed together with HRM from still videotape frames every 10 s for each work task (phases) with the OWAS method [6]. Using this method and *WinOWAS *software compulsive working postures were identified and necessary alterations were determined according to OWAS action categories: Category 1 = Normal postures, no action required; Category 2 = The posture is slightly harmful, actions to alter postures should be taken in the near future; Category 3 = The posture is distinctly harmful, actions to alter postures should be taken as soon as possible; Category 4 = The posture is extremely harmful, actions to correct postures should be taken immediately.

The rating of perceived exertion (RPE) of individuals depending on their age, physical conditions, subjective view of increased heart rate and muscle fatigue was also assessed using Borg rating scale, ranging from 6 to 20 [7,8]. Data were gathered via questionnaires and interviews.

Assessment of the functional state of skeletal muscle was carried out using myometric measurements with the *MYOTON*-3 device created in Estonia, University of Tartu [9]. The complete theoretical concepts of myotonometry (MYO) are described in references [10-12].

The principles of the MYO lies in using of acceleration probe to record the reaction of the peripheral skeletal muscle or its part to the mechanical impact and the following analysis of the resulting signal with the aid of the personal computer. Myoton exerts a local impact on the biological tissue by means of a brief impulse which is shortly followed by a quick release. The force of the impact is chosen such that it does not create changes in the biological tissue or precipitate the neurological reactions.

The criteria have been worked out enabling to contribute in the diagnostics of the functional condition of the skeletal muscles and correlate it with certain criteria of the classical diagnostics. Simultaneous measurements of intramuscular pressure (IMP), surface electromyography (SEMG) and MYO were investigated [12]. Time- and load-matched data revealed significant correlations between registered IMP, EMG and MYO parameters. The IMP and EMG amplitudes, as well as the MYO parameters (muscles frequency and stiffness) were linearly related to relative muscle load and a repeated measures using ANOVA analysis followed by determination of the intraclass correlation coefficient (ICC) determine reliability of MYO measuring. It was concluded that myotonometer is a reliable device for measuring skeletal muscle viscoelastic parameters; therefore, such electro-mechanical characterization of the skeletal muscle may provide new insights into muscle function and can help to diagnose the stage of pathological processes of muscles.

**Figure 1:**
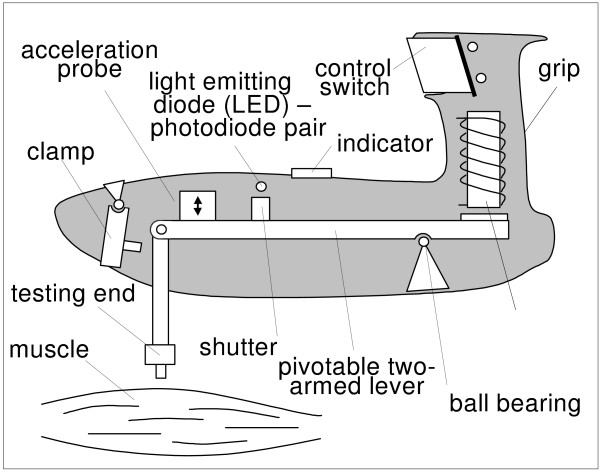
Schematic drawing of myotonometer.

**Figure 2:**
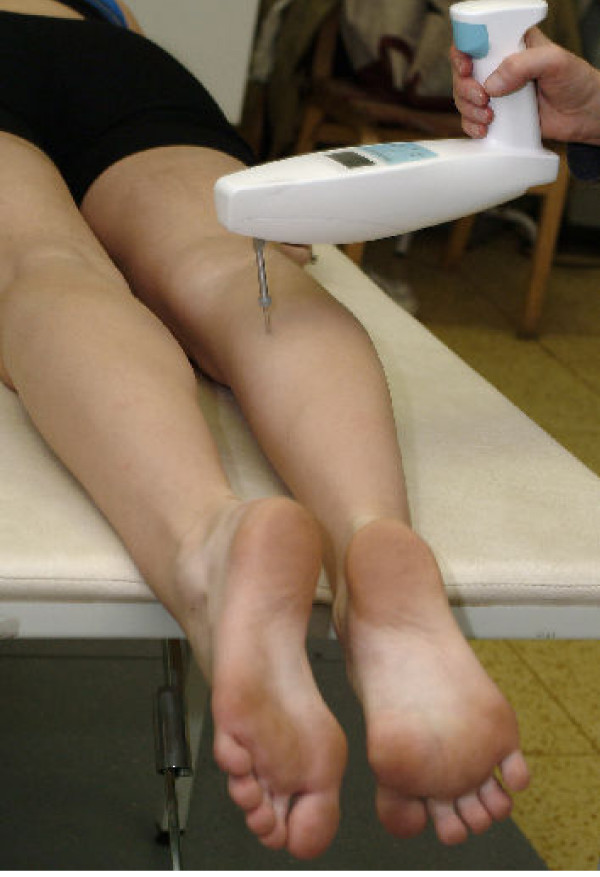
Myotonometrical testing of *m. gastrocnemius caput mediale*.

The testing end (mass 20 grams) of the computerized myotonometry (CMYO) device (Fig. 1) was in contact with muscle belly area (see Fig. 2), and the effective weight was employed on the surface of the measuring tissue. As a result, the tissues were in a compressed state. The CMYO device was fired in response to a fixed posture at the testing sensor end.

**Figure 3:**
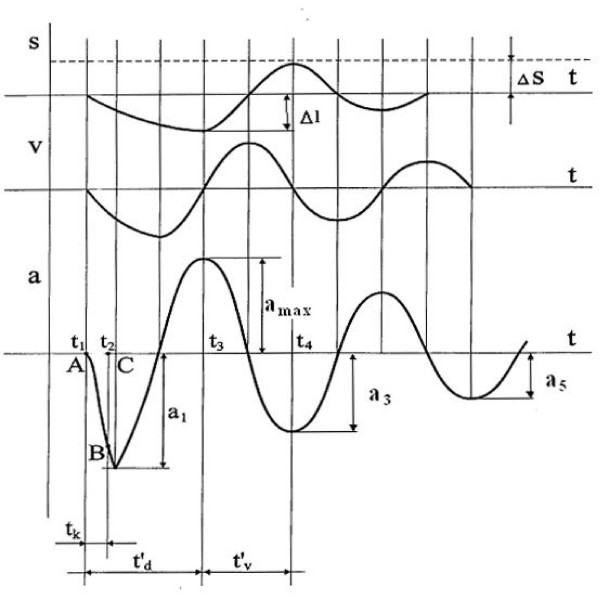
Waveforms of acceleration (a), velocity (v), and displacement (s), acquired in the process of damped natural oscillation performed by the myometer testing end.

The driver produced a short impulse (a few milliseconds, *t*_*k *_in Fig. 3), which was forwarded to the contact area. For our study, the duration of the impact on the muscle belly of studied muscles was 15 milliseconds. The force impulse terminated with a quick release at a moment *t*_2 _(Fig. 2). The tissue responded to the mechanical impact with damped oscillations. The oscillations were recorded by the acceleration transducer at the testing end of device.

The acceleration value of the first period of oscillations, calculated from the oscillation graph, characterizes the deformation of the tissue. The data of the next oscillation period provided the basis for calculating the oscillation frequency:



where ν is the oscillation frequency of the tissue, *T *– the oscillation period in seconds.

The logarithmic decrement of damping was calculated according to the following formulae:



where *Θ *is the oscillation logarithmic decrement of the tissue; *a*_3 _and *a*_5 _– the oscillation amplitudes.

The frequency of the damped oscillations measured during the rest period characterizes the tissue tone. The logarithmic decrement of the damped oscillations characterizes tissue elasticity. The decrement is inversely proportional to elasticity. Stiffness *C *reflects the resistance of tissue to the force that changes its shape and it was calculated by formula:



where *m *is the mass of the testing end of myometer; *a*_*max *_is the maximal amplitude of oscillation, and *Δl *is the depth of the displacement of the testing end.

Measurements for determination of muscle tone during one week work cycle were done with relaxed muscles before the work cycle had been started. In this way, one can obtain the most precise results when estimating muscle fatigue or the ability to restore elastic muscle qualities after the work cycle. Some data were also acquired showing the parameters of some muscle groups in the contracted state and reflecting their state during work.

Myotonometry testing of the following muscles was performed in relaxed state: *m*. *extensor digitorum*; *m*. *flexor carpi radialis*; *m. gastrocnemius *(*caput mediale*); *m. tibilais anterior *and *m. trapezius *(*upper part*). The procedure of muscle testing was performed in sitting position, on the table; the muscle length was middle; for all measurements the subject takes the same position.

### Statistical analysis

The results acquired were entered into the computer and processed using EXCEL software and statistical data processing program SPSS.11 according to popular descriptive statistical methods (Pearson's correlation coefficient *r*, a.o.). Reliability interval (interrater agreement) was also calculated determining Cohen's Kappa coefficient (*k*) [13]. This coefficient identifies connectivity of the experimental data, the number of participants and the proportion or correlation of the participants' acceptance of the experimental data:

*k *= (P_O _- P_C_)/(1 - P_C_),     (4)

where: P_O _– correspondence proportion of objective experimental data with respondents' responses (yes or no), P_C _– correspondence proportion of data with number of participants (P_C _= Σp_i_^2^, where p_i _is acceptance of each participant expressed in percent or as fractional number).

## Results

For HRM of road repair workers a 33 minutes long work period with following tasks was chosen: Task 1 – sand layer construction cycle 8 min and rest break 2 min; Task 2 – chipping layer construction cycle 10 min and rest break 5 min; Task 3 – asphalt layer construction cycle 8 min. Each task included different working phases (placing, profiling and leveling of the layer) which where investigated using OWAS analysis. The above mentioned tasks characterize a regularly repeated sidewalk repairing cycle. For pavers a 30 minutes long working cycle was chosen without a brake. Paving was divided into two phases. In the research following phases were analyzed: placing of sand layer – 5 min, paving 25 min. Research results of HRM, OWAS and RPE for selected teams are summed up in Tables 3 and 4.

**Table 3:** Workers' heart rate (HR), Pearson's correlation (*r*), Cohen's Kappa (*k*), percentage of the heart rate range (%HRR), energy expenditure (E), the rate of perceived exertion (RPE, scale 6–20), and work heaviness category (WHC). Road workers (n = 10), pavers (n = 10).

**Occupation**	**Heart rate monitoring**				**Mean %HRR ± SD**	**Mean E ± SD, kcal/min**	**Mean RPE ± SD (range)**	**WHC**
					
	**Mean HR ± SD, beats/min**	**Range HR, beats/min**	***r***	***k***				
Road workers	125 ± 14	108–160	0.95	0.68	52 ± 8	8,1 ± 1.5	15 ± 2 (13–17)	Hard work
Pavers	116 ± 13	82–150	0.92	0.59	42 ± 6	7.2 ± 1.1	12 ± 2 (11–13)	Moderate work

**Table 4:** Percent of workers whose muscle frequency exceeds the norm (> 16 Hz) after the work week cycle, Pearson's correlation (*r*), and Cohen's Kappa (*k*)

**Muscle groups**	**Road workers (n = 10)**			**Pavers (n = 10)**		
	
	**%**	***r***	***k***	**%**	***r***	***k***
*m. extensor digitorum*	60	0.78	0.34	28	0.68	0.59
*m. flexor carpi radialis*	60	0.78	0.21	30	0.70	0.35
*m. gastrocnemius*	25	0.82	0.50	80	0.80	0.54
*m. tibialis anterior*	50	0.85	0.35	90	0.75	0.50
*m. trapezius*	25	0.88	0.20	85	0.78	0.33

Computerized QWAS analysis showed that the most severe work phase for the road repair workers is carrying of the layer (sand, chip, asphalt) and is accordingly referable to Action Category (AC) 4. Profiling, in its turn, can be considered to be AC 2, while the levelling corresponds to AC 1. Acquired results for pavers show, that the phase 1 – spreading of sand before paving stones are put in place – can be characterized as AC 2, but the paving phase – as AC 3. The motivation will be described more particularly in the discussion part.

The above mentioned ergonomic analysis confirmed by the subjective statements of workers (using questionnaires and interviews). Inquiry data showed that the road maintenance workers most frequently complain on feeling discomfort after the work, specifically, fatigue or muscle pain in several parts of the body. After summing up the responses concerning pain or discomfort areas at the end of the workday, it was found out that the most workers complain on pain in their arms, legs, upper and lower part of the back, shoulder line. Although the work heaviness degree of road maintenance workers was identified, this study doesn't show the objective muscles fatigue. Therefore, a further research work was carried out using MYO measurements.

**Figure 4:**
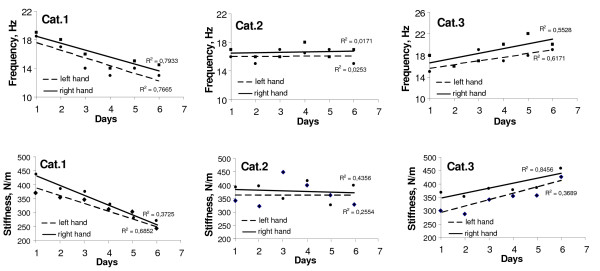
Results of the regression analysis of *m. extensor digitorum *frequency and stiffness during consecutive 6 work days in road workers.

According to regression analysis of MYO data, the slope of the lines (trendline) reflects the condition of the muscles after one week work cycle for all workers and can be subdivided into several categories (Fig. 4):

*Category I *– subject is able to relax the muscle;

*Category II *– muscle is able to adapt to the work load and to relax partly;

*Category III *– muscle is not able to relax.

**Figure 5:**
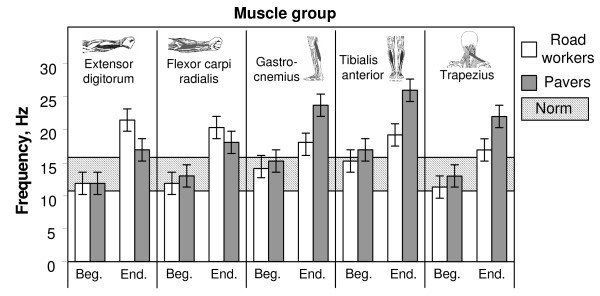
Illustration of frequency changing in separate muscle groups while performing the different kind of work at the beginning and at the end of the work week – for workers who are not to be able to adapt with the workload and whose muscle frequency exceeds the norm (11 up to 16 Hz, exist for each muscle individually) after the work week cycle.

Comparative data showing the load of separate muscle groups while performing different kinds of work are reflected in Fig. 5. The results show the average values of muscle frequency in the beginning and at the end of the work week, i.e. changes in the muscle tone and load for workers who are not to be able to adapt to the workload.

Tables 4 and 5 show the comparison of the different muscle groups which frequency and stiffness go beyond the norm after the work week cycle and percent of the workers having these changes. The percent of workers with differences in their muscle tone depending on the length of occupational service is shown in Table 6:

**Table 5:** Percent of workers whose muscle stiffness exceeds the norm (> 300 N/m) after the work week cycle, Pearson's correlation (*r*), and Cohen's Kappa (*k*)

**Muscle groups**	**Road workers (n = 10)**			**Pavers (n = 10)**		
	
	**%**	***r***	***k***	**%**	***r***	***k***
*m. extensor digitorum*	85	0.68	0.30	30	0.68	0.25
*m. flexor carpi radialis*	85	0.69	0.19	35	0.70	0.30
*m. gastrocnemius*	12	0.76	0.54	60	0.80	0.51
*m. tibialis anterior*	60	0.67	0.43	100	0.89	0.49
*m. trapezius*	25	0.55	0.25	90	0.78	0.28

**Table 6:** Percent of workers with differences in their muscle tone (categories I...III) after one week work cycle depending on the length of occupational service in the given road building company

**Occupation**	**Length of service in the occupation, years**								
	
	**1 – 5**			**5 – 10**			> **10**		
	
	**Category**	***r***	***k***	**Category**	***r***	***k***	**Category**	***r***	***k***
Road workers (n = 70)	I – 10%	0.68	0.48	I – 12%	0.68	0.45	I – 8%	0.68	12.0
	II – 13%	0.68	0.41	II – 17%	0.68	0.35	II – 12%	0.68	62.8
	III – 77%	0.68	0.53	III – 70%	0.68	0.25	III – 80%	0.68	89.3

Pavers (n = 25)	I – 14%	0.68	0.34	I – 10%	0.68	0.25	I – 11%	0.68	10.0
	II – 40%	0.68	0.33	II – 30%	0.68	0.52	II – 40%	0.68	18.1
	III – 46%	0.68	0.49	III – 60%	0.68	0.55	III – 49%	0.68	78.4

## Discussion

As we see from HRM data, the work heaviness degree for road repair workers and pavers is different, accordingly – hard work and moderate work. Interesting data were acquired observing decrease in the heart rate under conditions of different workload. By the length of a period when HR regained its normal state (as in rest periods) it is possible to assess approximately whether the worker has grown tired or has not. It was stated that HR of workers stabilizes (90–100 bets/min) when performing sand layer leveling with a rather low work intensity (2.5–3.1 kcal/min) and the HRM diagram shows "plateaus" area. Heart rate regains its normal state rather quickly – this process requires 5–8 min. Consequently – during this work phase the workers don't get tired. Whereas performing the work with a high intensity, hart rate increases constantly until it reaches 160 beats/min, sometimes – 170 or more beats/min, at the end of working cycle indicating that the worker is getting tired step by step. Also the period required for the heart rate to relax is longer – normal state is regained only in 30 minutes time.

In accordance with OWAS analysis also different Action Categories were identified. The heaviest work phase, as we have stated, is carrying of the layer (AC = 4). During this phase workers lift and move heavy loads – spades filled with sand, chippings or asphalt up to 10–12 kg (recommended weight limit calculated using NIOSH equations was 8 kg). Distance the heavy load has to be moved is from 2 to 20 meters or even more (depending on the possibility for the truck to drive closer to the place the layer should be constructed). Therefore, the posture is considered to be extremely harmful; actions to correct postures should be taken immediately. Also the posture for pavers (AC = 3) is considered to be distinctly harmful. Also in this case the actual frequently lifted mass (10 ± 2 kg) exceeds the weight limit up to 3 – 4 kg recommended by NIOSH. After having assessed individual work phases, it was found out that the most serious risks to the skeletal and muscular system of the workers are possible when heavy loads are lifted with stretched arms too high from the ground. For this reason, when thinking of work organization attention has to be paid to the factors making the performance of the work more difficult (e.g. high scaffolds, great distances to move heavy loads and the like). In this study training and muscle mass of the workers aren't considered, as well as the load and fatigue of individual muscles; these parameters are analyzed separately – by MYO investigations.

Analysis of the MYO data shows that for the road repair team the greatest load was put on arm muscles *m. extensor digitorum *and *m. flexor carpi radialis*. Road repair works usually involve fast movements of arms using spades, load on the shoulder line and relatively insignificant stiffness of calf-muscles. For example, very often two workers perform the construction of road edge (roadside) together; the construction of road edge requires lifting of a concrete edge which weights up to 100 kg. In this case, the normal frequency of muscles was exceeded for 60 per cent of workers, and the stiffness for – 85 per cent.

Work of the pavers is predominantly monotonous and most often is performed on knees or in a posture requiring bending, thus especially contracting muscles of both legs and shoulders. It was justified by MYO data – the greatest load for pavers was put on their leg muscles, especially *m. tibialis anterior *and *m. trapezius *of shoulders (see Fig. 5). Both the frequency and stiffness were beyond the normal condition for 90 per cent of workers. The increase of the leg muscle tone can be explained by an increased static load on the legs. However, for arm muscles with an increased stiffness, the frequency goes beyond the norm (determinable for each muscle individually) only for 28 per cent of workers (*m. flexor carpi radialis*). Tension which depends on stiffness parameters of *m. extensor digitorum *and *m. flexor carpi radialis *for the pavers is smaller than for the road repair team workers. This results from the fact that the weight the pavers work with is smaller. Usually they lift a paving stone with the average weight of 5 to 6 kg (in some cases it could be 10 kg) with both hands, put it on the sand layer and using a hand mattock-hammer, whose weight is less than 0.5 kg, fix the stone in its proper place. The most common movements are done by one arm (usually the right arm) while smoothing sand and hitting the paving stone are done with the mattock-hammer.

MYO data also show that muscles, which are located in different sides of the body, are adapted to work load differently. It was stated both for road workers and for pavers. Differences in the load of both arm and leg muscles are significantly different. There are some workers working with both arms equally, for some others the left arm is involved more than the right. However it doesn't mean that these workers are left-handed; the only reason for this is the specificity of the performed activity, namely, the weight of a material, which has to be carried on the spade held by the left arm.

The proportion of workers with differences in their muscle tone depending on the length of service in the specific occupation was various. As we see, the number of road workers with an increased muscle tone (Category III) increases when the length of service is more than 10 years und reaches in this case 80 per cent. The greatest number of pavers (60 per cent) falling within Category III is after 5 to 10 years of service.

MYO data showed that office employee's leg muscles are loaded, too. It was an unexpected finding even for the office employees themselves. However, many of them used while working foot stands thus tensing *m. tibialis anterior *(with toes uplifted) or worked in other postures with tensed leg muscles. These employees have greater differences for their legs. As it was observed office employees sometimes don't put the whole foot on the floor, but support one or both legs only on their toes or keep their feet on the horizontal bars of their chairs thus straining also *m. gastrocnemius*. Tone of these muscles, when they are contracted for a longer period, increases, thus making disorders of blood circulation and herewith also muscular skeletal system (MSS) diseases possible.

Determination of muscle stiffness and frequency is of great importance, for fatigue can be subdivided into high-frequency fatigue (HFF) and low-frequency fatigue (LFF), what differs from mechanic (also electric) features of muscles. HFF fatigue is characterized by an excessive loss of force at high frequencies of stimulation and rapid recovery when the frequency is reduced. Frequencies in excess of 50 Hz are rarely observed by voluntary activation of human muscle, and for this reason there has been some doubt as to whether high-frequency fatigue is a significant feature of normal activity. Recent experiments have shown that with 30 Hz stimulation there is a more rapid loss of force if the muscle is held isometric in a shortened position and the fatigue is rapidly reversed if the muscle is re-extended, even under ischemic conditions [14]. These findings are consistent with the accumulation of K^+ ^in the t-tubules and interfibre spaces of the muscle. LFF is characterized by a relative loss of force at low frequencies of stimulation and a slow recovery over the course of hours or even days and there is evidence provided by intracellular measurements that low-frequency fatigue is a result of reduction in Ca^2+ ^release.

It must be noted that by summing up the results on muscle tone and fatigue acquired during the research work, our main interest was concentrated on the factors that allowed some muscle groups to relax and restore capacity to work. Data acquired during research (see subdivision of workers into 3 categories according to MYO data) are indicative of several features of HFF and LFF.

Features of HFF:

- Force of muscles is restored quickly after the irritation frequency has decreased, especially what concerns workers falling within category I. This concerns partly also those whose muscle tone remains unchanged during their work (in this case – during one week work cycle) – category II.

Features of LFF:

- Greater decrease of contraction force as in the case of HFF;

- Force restores slowly, during several hours, but in some cases total restoration occurs only in several days.

It has been observed that for several persons working in the road repair team, physiological LFF of muscles (this type of fatigue is called also – lasting fatigue) is accompanied by pain. If the work load requires too much muscle loading and stretching, when eccentric contractions are created, active muscle fibres resist the stretching and the cause of pain is the ultra-structural damage of the muscle.

For some workers muscle tone remained the same the whole week through, which means that they were able to adapt to the existing work load. It has to be noted that for the most of the workers MYO parameters after rest on Saturday and Sunday had decreased again. However, for some workers parameters remained relatively high, because in their days off, they did some other kind of hard physical work.

Consequently the road construction sphere requires special rest exercises to be developed and this is the task of ergonomists and occupational doctors.

## Conclusion

According to the heart rate monitoring data the road construction and maintenance workers – road repair workers and pavers – can be subdivided into hard and moderately hard work categories and despite rapid technical improvements, their work still requires hard manual labor, compulsive working postures and constantly repeated arm movements. We conclude that road construction and maintenance workers can be subdivided into three categories basing on the dynamics of muscle tone during one work week, and complex analysis – consisting of heart rate monitoring, compulsive working postures assessment and myotonometry is appropriate to assess the degree of work heaviness – may provide prognosis of occupational pathology or work-related musculoskeletal disorders for the workers under different workload conditions and can be also used when choosing necessary rest time and its periodicity.
